# A new *Notomastus* (Annelida, Capitellidae) species from Korean waters, with genetic comparison based on three gene markers

**DOI:** 10.3897/zookeys.754.23655

**Published:** 2018-05-04

**Authors:** Man-Ki Jeong, Ho Young Soh, Jin Hee Wi, Hae-Lip Suh

**Affiliations:** 1 The Fisheries Science Institute, Chonnam National University, Daehak-ro, Yeosu 59626, South Korea; 2 Faculty of Marine Technology, Chonnam National University, Daehak-ro, Yeosu 59626, South Korea; 3 School of Environmental Science and Engineering, Gwangju Institute of Science and Technology, Choemdangwagi-ro, Buk-gu, Gwangju 61005, South Korea; 4 Department of Oceanography, Chonnam National University, Yongbong-ro, Buk-gu, Gwangju 61186, South Korea

**Keywords:** Polychaeta, *Notomastus koreanus* sp. n., morphology, DNA barcoding, COI, 16S rRNA, histone H3, South Korea

## Abstract

*Notomastus koreanus* **sp. n.**, collected from the sublittoral muddy bottom of Korean waters, is described as a new species. The Korean new species closely resembles *N. torquatus* Hutchings & Rainer, 1979 in the chaetal arrangement and the details of abdominal segments, but differs in the position of genital pores and the absence of eyes. DNA sequences (mtCOI, 16S rRNA, and histone H3) of the new species were compared with all the available sequences of *Notomastus* species in the GenBank database. Three genes showed significant genetic differences between the new species and its congeners (COI: 51.2%, 16S: 38.1–47.3%, H3: 3.7–9.3%). This study also includes a comprehensive comparison of the new Korean *Notomastus* species with its most closely similar species, based on the morphological and genetic results.

## Introduction

Capitellid polychaetes build spiral burrows or U-shape tubes in bottom sediments, which increase the subsurface penetration of water and oxygen, thus improving the recruitment and growth of small benthic organisms (Fauchald and Jumars 1979, Scaps 2002). In particular, the genus *Notomastus* Sars, 1851 is one of the most common and species-rich genus in the Capitellidae Grube, 1862 and occurs from the intertidal to the deep sea in a variety of sediment types including fine, medium, and silty sand and mud (Dean 2001). It currently contains 43 valid species, which is the highest number of species among the capitellid genera (Gil and Bellan 2017). Despite their ecological success and high species diversity, the lack of good generic characters and the incorrect descriptions in several previous records have led to taxonomic confusion in the genus (Green 2002). For instance, the hooded hook dentition of *N. latericeus* Sars, 1851 has been described differently in the published records of the species, and the protruded lateral organs had been mistaken as the branchiae in the former records of *Notomastus* species from Japan and Vietnam (Day 1967, Fauvel 1927, Green 2002, Thomassin 1970).

The taxonomic boundary of the genus *Notomastus* has been continually modified over the last century. The genus was designated by Sars (1851) with the description of the type species, *N. latericeus*. Eisig (1887) divided *Notomastus* into two subgenera, *Tremomastus* and *Clistomastus*, by the presence/absence of genital pores in the abdomen and the development of hooded hooks. Fauvel (1927) suggested that the subgeneric name of Notomastus (Notomastus) should replace Notomastus (Tremomastus) and Hartman (1947) accepted this view. However, Day (1967) and Fauchald (1977) did not agree with these subgeneric categories in their diagnoses of the genus. Ewing (1982) placed three genera, *Dodecaseta* McCammon & Stull, 1978, *Paraleiocapitella* Thomassin, 1970, and *Rashgua* Wesenberg-Lund, 1949, within *Notomastus*. Green (2002) clarified that *Dodecaseta* and *Rashgua* differed from *Notomastus* in the chaetal distribution, which was regarded as a good generic character. Green (2002) also suggested the need for a review of the taxonomic boundary of *Notomastus* and its species. In this study, *Notomastus* is defined based on the characteristics of its 12 thoracic segments, which comprise an achaetigerous peristomium and 11 chaetigers, including a uniramous or biramous first chaetiger, subsequent chaetigers usually with only capillaries, and posterior thoracic chaetigers with capillaries and sometimes neuropodial hooks; abdominal segments have only hooks. Although this study provides detailed descriptions of the *Notomastus* species from Korean waters, the comparison with closely related species was limited due to the insufficient morphological information of many records. To overcome this difficulty, studies using a combination of morphological analysis and DNA barcoding have been conducted to distinguish closely related capitellid species and to improve species recognition between them (Jeong et al. 2017b, Silva et al. 2016). The aim of the present study is to clarify the taxonomic status of the undescribed *Notomastus* species of Korea by morphological and genetic analysis using three different partial genes (mtCOI, 16S rRNA, and H3) and to compare Korean species with their closest congeners.

## Materials and methods

**Figure 1.**
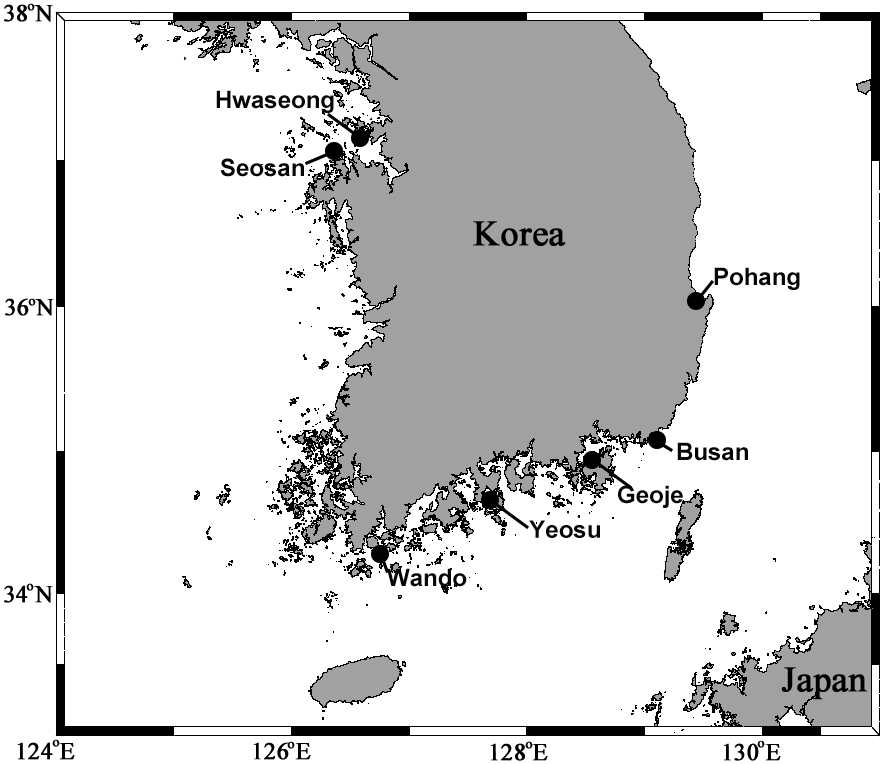
Map of study area with main collecting locations.

**Morphological analysis.** Samples were collected from seven stations in sublittoral areas of Korea using a 0.05 m^2^ Van Veen grab (Fig. 1). The sediment samples were elutriated over a 0.5 mm sieve in a 30 l seawater container, and the organisms were transferred to a 1 l collecting jar with 7% MgCl_2_ solution for anesthesia. The relaxed samples were fixed in a buffered solution of 10% formalin within 2 hours and finally preserved in 90% ethanol. In the laboratory, *Notomastus* specimens were sorted under a stereomicroscope (SMZ745T, Nikon). Line drawings were generated using a differential interference contrast microscope (Eclipse Ci-L, Nikon) and a digital pen display (Cintiq 22HD, Wacom). Methyl green staining patterns (MGSP) and scanning electron microscopy (SEM) analyses were described and photographed, as delineated by Jeong et al. (2017b). The examined type materials were deposited in the collections of the Marine Biodiversity Institute of Korea (MABIK) in Seocheon, Korea (Table 1). Two additional specimens (voucher numbers: NIBRIV0000634919 and NIBRIV0000634920) were deposited at the National Institute of Biological Resources (NIBR) in Incheon, Korea.

**Table 1.** A list of sampling localities, species names, sample types, voucher numbers, Genbank accession numbers, and references.

Location		Latitude / Longitude (DDM)	Species name	Type	Voucher number	Accession number of Genbank			References
						mtCOI	16SrRNA	histone H3	
South Korea	Yeosu	34°39.03'N, 127°40.86'E	*N. koreanus* sp. n.	Paratype	NA00146048	MG437146	MG748697	MG748700	This study
				Paratype	NA00146049	MG437147	MG748698	MG748701	
	Busan	35°5.83'N, 129°2.42'E		Paratype	NA00066329	MG437148		MG748699	
		35°6.33'N, 129°3.31'E		Holotype	NA00066337				
	Hwaseong	37°8.95'N, 126°35.39'E			NA00066302		MG748696		
	Geoje	34°54.17'N, 128°36.98'E		Paratype	NA00066311				
	Pohang	36°1.31'N, 129°25.16'E		Paratype	NA00066396				
Portugal	Sado estuary	38°29.22'N, 8°53.1'W	*N. profondus*		RR132	KR916897			Lobo et al. (2016)
Canada	Bamfield		*N. hemipodus*				HM746714	HM746759	Paul et al. (2010)
Sweden	Bohuslän		*N. latericeus*		SMNH75827		AY340469	DQ779747	Rousset et al. (2007)
Australia			*N. torquatus*		AMW23426			AF185258	Brown et al. (1999)
China	Bohai Sea	38°21.12'N, 120°7.92'E	*Notomastus* sp.		BIOUG03550-A09				BOLD Systems (2017)

**Molecular analysis.** Genomic DNA was extracted from tissue obtained from partial dissection of the middle part of the abdomen of the ethanol-preserved specimens. To extract the genomic DNA, 1.5 mL centrifuge tubes each containing 90 μL of 10% Chelex suspension (Bio-Rad Laboratories Inc.), 10 μL of Proteinase K (10 mg/ml, iNtRON Biotechnology, Inc.) and dissected tissues (ca. 1/2 segment) were incubated at 56 °C for 3–12 hours.

The extracted genomic DNA was used as a template to amplify the target region. Polymerase chain reaction (PCR) was performed on a MasterCycler PCR thermal cycler (Eppendorf Co.). The primer pair for COI was LoboF1 and LoboR1 (Lobo et al. 2013), for 16S rRNA was 16SarL and 16SbrH (Palumbi 1996) and for histone H3 was H3F and H3R (Colgan et al. 1998). The PCR mixtures contained 16 μL of deionized water, 1 μL of each primer (10 μM), 2 μL of DNA template and PCR premix (BiONEER Co.). The temperature profile was as follows: 94 °C/180s–(94 °C/30s–48 °C/30s–72 °C/60s)*40 cycles–72 °C/420s for mtCOI, 94 °C/180s–(94 °C/45s–50 °C/60s–72 °C/60s)*35 cycles–72 °C/420s for 16S rRNA, and 94 °C/180s–(94 °C/45s–50 °C/60s–72 °C/60s)*35 cycles–72 °C/420s for histone H3. The results of the PCR amplification were confirmed on 1.0% agarose gels using ethidium bromide staining. Purification and sequencing of the obtained PCR products were performed at the Macrogen Inc. facilities (Seoul, Korea).

The forward and reverse sequences were compared and edited using Chromas software version 2.3 (Technelysium Pty. Ltd.). The partial sequences of the COI, 16S rRNA and H3 genes were aligned with the sequences of available *Notomastus* species obtained from GenBank (https://www.ncbi.nlm.nih.gov/genbank/) using the Molecular Evolutionary Genetics Analysis (MEGA) software version 7.0 (Kumar et al. 2016). Table 1 summarizes information for all sequences used in the analyses. The aligned sequences were used as data sets to generate the genetic distance using Kimura’s two-parameter (K2P) model (Kimura 1980). Based on the K2P distances, we calculated the intraspecific genetic differences within the Korean specimens and the interspecific genetic differences among the closest taxa.

## Results

### Systematics

#### Family Capitellidae Grube, 1862

##### Genus Notomastus

Taxon classificationAnimaliaCapitellidaCapitellidae

Sars, 1851

###### Type species.

*Notomastus latericeus* Sars, 1851

###### Type locality.

Komagfjord, Norway

###### Generic diagnosis

(modified after Green 2002). Thorax with 12 segments including an achaetous peristomium and 11 chaetigers with capillary chaetae. Last three thoracic chaetigers may have capillary chaetae in both rami or may be transitional with capillary chaetae in notopodia and hooded hooks in neuropodia. Remaining chaetigers with hooded hooks only. Hooded hooks with one or more rows of teeth above main fang; more than two teeth in basal row. Branchiae may be present or absent.

###### Remarks.

According to the former generic diagnosis by Green (2002), *Notomastus* may or may not have a transitional chaetiger with capillary notochaetae and neurohooks in the last part of thorax. However, *N. precocis* Hartman, 1960 and *N. teres* Hartman, 1965 have three and two transitional chaetigers in the posterior thoracic region, respectively (Gil and Bellan 2017, Hartman 1960, 1965). Therefore, the generic diagnosis was amended including the expanded range of the thoracic chaetal arrangement.

##### Notomastus koreanus sp. n.

Taxon classificationAnimaliaCapitellidaCapitellidae

http://zoobank.org/18FE9853-2A6B-45B4-9C79-E3E7569C9E3B

Figs 2A–D , 3A–G

###### Materials examined.

Holotype: MABIKNA00066337, sex uncertain, Busan, 35°6.33'N, 129°3.31'E (DDM), subtidal, sandy mud bottom, 16 m depth, October 2011, collected by Byoung-Mi Choi. Paratypes: MABIKNA00146048, MABIKNA00146049, sex uncertain, Yeosu, 34°39.03'N, 127°40.86'E, subtidal, sandy mud bottom, 20 m depth, October 2017, collected by Man-Ki Jeong; MABIKNA00066329, sex uncertain, Busan, 35°5.83'N, 129°2.42'E subtidal, sandy mud bottom, 15 m depth, October 2011, collected by Byoung-Mi Choi; MABIKNA00066396, sex uncertain, Pohang, 36°3.09'N, 129°23.55'E, subtidal, sandy mud bottom, 12 m depth, January 2012, collected by Byoung-Mi Choi; MABIKNA00066311, sex uncertain, Geoje, 34°54.17'N, 128°36.98'E, subtidal, sandy mud bottom, 10 m depth, January 2012, collected by Byoung-Mi Choi.

###### Additional materials examined.

MABIKNA00115263, sex uncertain, Busan, 35°4.7'N, 128°55.4'E, subtidal, sandy mud bottom, 14 m depth, January 2012, collected by Byoung-Mi Choi; MABIKNA00066302, sex uncertain, Hwaseong, 37°8.95'N, 126°35.39'E, subtidal, sandy mud bottom, 20 m depth, September 2011, collected by Byoung-Mi Choi; MABIKNA00066303, MABIKNA00115303, sex uncertain, Seosan, 37°2.03'N, 126°23.94'E subtidal, sandy mud bottom, 15 m depth, September 2011, collected by Byoung-Mi Choi; MABIKNA00066385, sex uncertain, Pohang, 36°1.31'N, 129°25.16'E subtidal, sandy mud bottom, 12 m depth, November 2010, collected by Byoung-Mi Choi; MABIKNA00115314, sex uncertain, Wando, 34°22.12'N, 127°0.79'E, subtidal, sandy mud bottom, 10 m depth, September 2011, collected by Byoung-Mi Choi. Additional 3 specimens from type locality on SEM stub.

###### Diagnosis.

Thorax with achaetigerous peristomium and 11 chaetigers. Anterior 5 thoracic segments tessellated. First chaetiger without neuropodia. Chaetigers 1–11 with capillary chaetae only. Abdominal chaetigers with hooded hooks only. Lateral organs not protruded above surface, narrow and oval shape, present along body. Genital pores present in intersegmental furrows between chaetigers 7–8, 8–9, 9–10, and 10–11. Parapodial lobes in anterior to moderate abdominal region not protruded. Posteriorly extended parapodial lobes present on posterior abdominal segments. Pygidium without anal cirri.

###### Description.

Holotype entire, about 80 mm long, 1.2 mm wide for 280 chaetigers. Paratype material ranges from 31–87 mm in length, 0.7–1.3 mm width with 30–270 chaetigers. Body elongate, rounded dorsally, flattened ventrally, widest in anterior thoracic chaetigers, with ventral white line in abdominal region. Color in alcohol whitish yellow.

**Figure 2.**
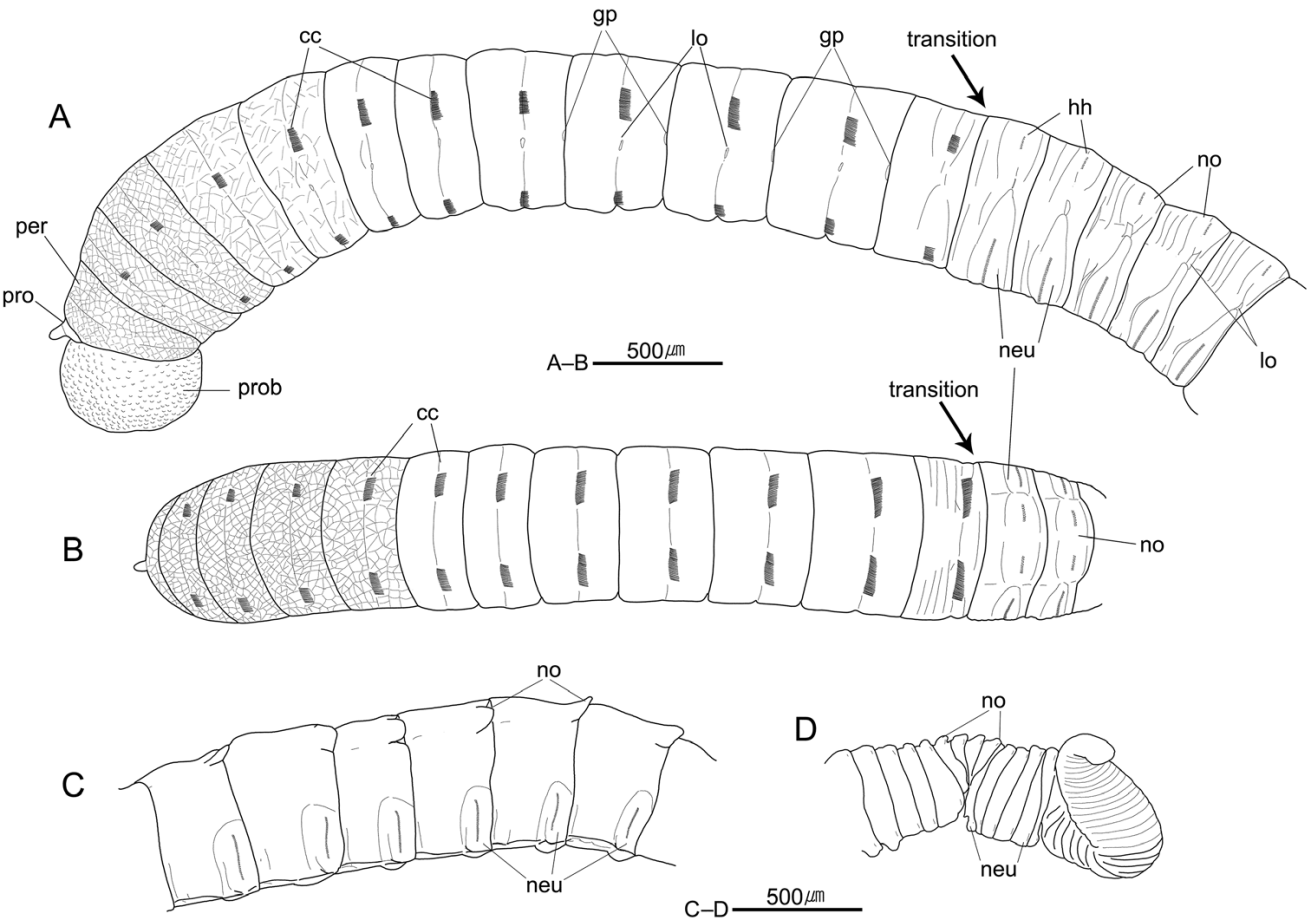
*Notomastus koreanus* sp. n. **A** anterior end, left lateral view (MABIKNA00146048) **B** same, dorsal view (MABIKNA00146048) **C** posterior abdominal segments, left lateral view (Holotype, MABIKNA00066337) **D** posterior end, left lateral view (Holotype, MABIKNA00066337). Abbreviations: cc, capillary chaetae; gp, genital pore; hh, hooded hooks; lo, lateral organ; neu, neuropodium; no, notopodium; pro, prostomium; prob, proboscis; per, peristomium; transition, transition between thorax and abdomen.

**Figure 3.**
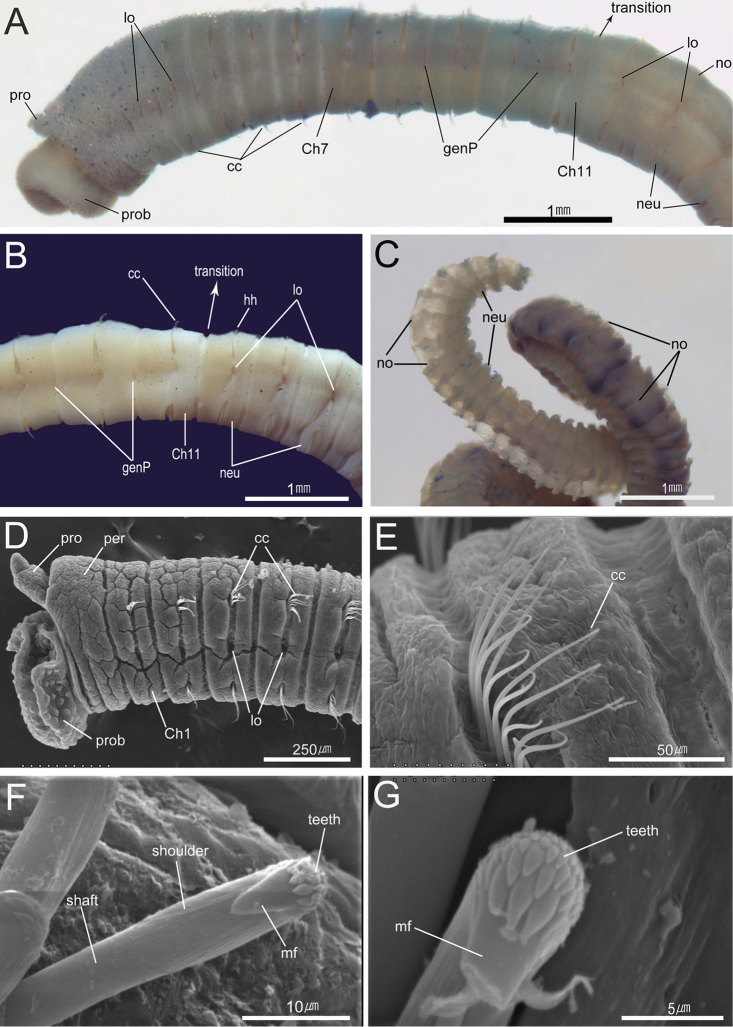
*Notomastus koreanus* sp. n. **A−C** photomicrographs **A** anterior end in left lateral view (showing methyl green staining reaction, MABIKNA00066311) **B** chaetigers 9–15 in left lateral view (MABIKNA00066396) **C** posterior end (MABIKNA00066396) **D–G** scanning electron micrographs (using additional specimens from type locality) **D** anterior 6 thoracic segments in left lateral view **E** capillary chaetae of chaetiger 4 **F–G** abdominal hooded hooks in frontal view. Abbreviations: cc, capillary chaetae; Ch, chaetiger; genP, genital pore; hh, hooded hooks; lo, lateral organ; mf, main fang; neu, neuropodium; no, notopodium; per, peristomium; pro, prostomium; prob, proboscis; transition, transition between thorax and abdomen.

Prostomium conical, with short and rounded palpode; nuchal organs not seen, eyespots absent (Figs 2A, 3A, D). Proboscis everted, with numerous hemispherical papillae (Figs 2A, 3D). Peristomium achaetous, weakly biannulated, slightly longer than first chaetiger (Figs 2A, 3D).

Thorax with 12 segments including achaetous peristomium and 11 chaetigers (Figs 2A–B, 3B). Thoracic segments biannulated with intra- and inter-segmental furrows (Figs 2A–B, 3A). Anterior thoracic segments tessellated; peristomium and chaetigers 1–2 tessellated, chaetigers 3–4 slightly tessellated; remaining segments smooth (Figs 2A–B, 3D). First chaetiger with only notopodia having 12 capillaries per fascicle; remaining thoracic chaetigers with 40–60 bilimbate capillaries per fascicle in both parapodia (Figs 2A–B, 3D–E). Thoracic parapodia reduced, located in intra-segmental furrows (Figs 2A–B, 3D); notopodia dorso-laterally on first chaetiger and middorsally on following chaetigers (Fig. 2B); neuropodia ventrolaterally on whole chaetigers. (Figs 2A, 3A–B). Lateral organs not protruded above surface, narrow and oval shape, present along body, situated in furrow between notopodia and neuropodia, less distinct in posterior abdominal region (Figs 2A–B, 3A–D); position of lateral organs slightly nearer to neuropodium in chaetigers 1–3, nearer to notopodium in following chaetigers (Figs 2A, 3A, D). Genital pores present in intersegmental furrows of between chaetigers 7–8, 8–9, 9–10, and 10–11 (Figs 2A, 3A–B).

Transition between thorax and abdomen distinguished by changes in shape of chaeta and segment (Figs 2A–B, 3A–B); last thoracic chaetiger bi-annulated, with capillaries only, slightly thinner than first abdominal chaetiger; anterior abdominal segments multi-annulated, with better developed neuropodial lobes than thoracic ones, having hooded hooks only (Figs 2A–B, 3B). Parapodia in anterior to mid abdominal region not protruded, well separated (Figs 2A–B, 3A–B). Notopodial lobes not protruded in anterior abdomen, middorsal on anterior few segments, becoming dorsolateral in following abdominal region, with 6–15 hooded hooks only per fascicle, having posteriorly extended and semicircular lamella from chaetiger 160 to end of body (Figs 2A–C, 3A–C). Neuropodial lobes having 15–30 hooded hooks per fascicle, well separated and weakly protruded in anterior abdomen, more protruded and almost fused ventrally in posterior abdomen, partially fused to notopodial lobes in posterior end (Figs 2A, C, 3A–C); dorsal tips of neuropodial lobes do not protruded above surface, extended below lateral organs in anterior to mid abdominal region (Figs 2A, C, 3B).

Hooded hooks with main fang extending slightly beyond hoods; hood slightly flared. Main fang of hooded hooks with 3 rows of small teeth; 5 in basal row, 6–8 in second row, and at least 6 in superior row (Fig. 3F–G).

Digitiform branchiae not observed in abdomen; each notopodial lobe with posteriorly extended semicircular lamella in posterior abdomen (Figs 2C, 3C). Pygidium simple, without anal cirri (Fig. 2D).

###### Methyl green staining pattern.

Anterior thoracic segments (peristomium and chaetigers 1–6) not stained. Posterior thoracic segments (chaetigers 7–10 or 11) stained (Fig. 3A); chaetiger 10 and dorsum of chaetiger 7 more deeply stained with blue (Fig. 3A). Anterior few abdominal chaetigers temporary stained with green; fading within 10 minutes (Fig. 3A). Ventral side of abdominal segments having pair of longitudinal green bands. Individual-specific variations observed; sometimes chaetigers 5–6 and chaetiger 11 weakly stained with blue, posterior edge of each abdominal segment stained with blue in large specimens (Fig. 3C).

###### Etymology.

The new species is named for its wide distribution in coastal waters of Korea.

###### Distribution.

The subtidal areas (10–20 m) near Korea (Fig. 1). The subtidal habitat (ca. 20 m) of Bohai Sea, China (see details in Discussion).

###### Ecology.

*Notomastus koreanus* sp. n. was sampled from soft sediments throughout the year. Most well-developed individuals (having over 250 segments) were obtained between October and January. The sediment of the collecting stations was mainly composed of sandy mud with shell fragments. *Leiochrides yokjidoensis* Jeong, Wi & Suh, 2017 and an undescribed *Heteromastus* Eisig, 1887 species co-occurred in southern stations of this study.

###### Remarks.

*Notomastus koreanus* sp. n. is distinguished from other species of the genus by the morphological combination of absence of distinct eyes and first neuropodia, last thoracic chaetigers with only capillary chaetae, presence of genital pores between chaetigers 7–11, non-protruded lateral organs and neuropodial lobes in anterior abdomen, and posteriorly extended parapodial lobes in posterior abdomen. The new Korean *Notomastus* species closely resembles *N. torquatus* Hutchings & Rainer, 1979 in the chaetal arrangement, the absence of developed neuropodial lobes in anterior abdomen, and the presence of posteriorly extended parapodial lobes in the posterior abdomen (Table 2). However, they differ in the presence of eyes on posterior prostomium (eyespots vs. absence) and the location of genital pores (between chaetigers 3 or 5–10 vs. 7–11, Table 2). Additionally, *N. torquatus* is regarded as an endemic species of Australia and has a much wider thorax (4 mm vs. 1.3 mm) than comparable specimens of *N. koreanus* sp. n., which have 280 segments when fully developed (Doyle 1991, Hutchings and Rainer 1979). *Notomastus hemipodus* Hartman, 1945 and *N. tenuis* Moore, 1909 are also similar to *N. koreanus* sp. n. in the chaetal arrangement and the absence of protruded neuropodial lobes in anterior abdomen, but clearly differ in the details of the eyes, the genital/lateral organs, and the MGSP (Table 2). Moreover, they have the unique features of the indistinct palpode and the bilobed notopodial lobes, respectively.

**Table 2.** Morphological comparison between Korean *Notomastus* species and its closely similar species. A: absent; P: present; Ch: chaetiger; NM: not mentioned; abd: abdomen; th: thorax; uni: uniramous; bi: biramous.

Species	First Ch	Eyes	Distinct palpode	Lateral organs	Genital pores	Dental structure of hooks	Parapodial lobes in posterior abdomen	Methyl green staining patterns	Habitat (locality)	References
*N. koreanus* sp. n.	uni	A	P	not protrude	between Ch 7–11	>17 teeth in 3 rows (5/6–8/>6)	parapodial lobes posteriorly extended	dorsum of Ch 7 and Ch 8–10 stained blue, Ch 5–6 and 11 sometimes stained, abd with 2 ventral blue lines	subtidal, 10–20 m, sandy mud with shell fragments (Korea)	This study
*N. latericeus*	bi	P	P	not protrude	between Ch 7–20	3 rows (5/?/?)	not extended	NM	intertidal to abyssal, sand, mud (cosmopolitan)	Day 1967
*N. hemipodus*	uni	P (single pair)	P	protrude on anterior abd	between Ch 8–12	16–24 teeth in 3 rows (4–6/6–8/6–8)	bilobed notopodial lobes posteriorly extended	Ch 1–6 green, Ch 7–10 blue, dorsum of abd green, abd with 2 ventral blue lines	intertidal to shelf depths, 0.5–426 m (America)	García-Garza et al. 2012, Green 2002
*N. tenuis*	uni	P (eyespots)	A	protrude on anterior abd	between Ch 5–10	many teeth (NM) in 4–5 rows	notopodial lobes posteriorly extended	Whole segments stained with light green	intertidal to shallow subtidal (America)	García-Garza et al. 2012
*N. torquatus*	uni	P (eyespots)	P	NM	between Ch 3 or 5– 10	16–24 teeth in 3 rows (4–6/6–8/6–8)	parapodial lobes posteriorly extended	NM	sea grass beds on muddy sand (Australia)	Hutchings and Rainer 1979

###### Genetic comparison with the published sequences of *Notomastus* species.

To confirm the genetic distances among the new species and its closely related species, we used the partial sequences of mitochondrial (mtCOI and 16S rRNA) and nuclear (histone H3) genes. In all genetic comparisons, the intraspecific differences among the Korean specimens were negligible (0–0.1%, Table 3). The mean interspecific differences for mitochondrial COI (50.9%) and 16S rRNA (43.2%) genes were much higher than the mean interspecific difference for the nuclear histone H3 gene (7.6%). In the mtCOI gene comparison, the mean genetic difference between *N. koreanus* sp. n. and *N. profondus* (Eisig, 1887) of Portugal (KR916899) was substantial (51.2%, Table 3). In the interspecific comparison for the 16S rRNA gene, *N. koreanus* sp. n. was well distinguished from *N. hemipodus* (38.1%, HM746714) of Canada and *N. latericeus* (47.3%, AY340469) of Sweden (Table 3). In the histone H3 gene comparison, *N. koreanus* sp. n. genetically differed from *N. torquatus* (3.7%, AF185258) of Australia, *N. latericeus* (7.0%, DQ779747) of Sweden, and *N. hemipodus* (9.3%, HM746759) of Canada (Table 3). Previously known genetic difference of the mtCOI and the 16S rRNA genes among the capitellid species is generally about 18–20% (Jeong et al. 2017b, Silva et al. 2016). In contrast, the histone H3 gene difference between cryptic nereidid polychaetes is around 2–9% (Glasby et al. 2013). Thus, the genetic differences between *N. koreanus* sp. n. and its closely related species (COI: 51.2%, 16S: 38.1–47.3%, H3: 3.7–9.3%) is significant at the species level revealing the speciation among them. On the other hand, the mtCOI gene sequence of the Chinese specimen (BIOUG03550-A09, Table 1) is genetically matched with *N. koreanus* sp. n. (0.007 in K2P distance, Table 3), although it has been reported as *N. latericeus* on BOLD (www.barcodinglife.org) database (BOLD Systems 2017). *Notomastus latericeus* was originally described from Norwegian waters, and it is easily discriminated from our new species in terms of morphology (Table 2). The published histone H3 and 16S rRNA sequences of *N. latericeus* from Swedish waters are clearly distinguished from the sequences of *N. koreanus* sp. n. by the significant genetic difference (Table 3). Thus, the mtCOI sequence of the Chinese specimen on the BOLD database is regarded as a misidentification at the species level and confirms the additional occurrence of our new species in the Bohai Sea of northeastern China.

**Table 3.** Mean genetic distances between examined *Notomastus* species based on K2P distance. Bold numbers represent the mean intraspecific K2P distance of Korean specimens.

mtCOI	1	2	3
1. *N. koreanus* n. sp. (Korea)	**0.001**		
2. *Notomastus* sp. (China)	0.007	–	
3. *N. profondus* (Portugal)	0.512	0.506	–
**16S rRNA**	1	2	3
1. *N. koreanus* n. sp. (Korea)	**0.000**		
2. *N. hemipodus* (Canada)	0.381	–	
3. *N. latericeus* (Sweden)	0.473	0.441	–
**histone H3**	1	2	3
1. *N. koreanus* n. sp. (Korea)	**0.000**		
2. *N. torquatus* (Australia)	0.037	–	
3. *N. latericeus* (Sweden)	0.070	0.088	–
4. *N. hemipodus* (Canada)	0.093	0.092	0.075

#### Key to species of *Notomastus* closely similar to the Korean new species.

**Table:** 

1	First chaetiger biramous; dorsally protruded neuropodial lobes present in anterior abdomen; genital pores present between chaetigers 7–20	***N. latericeus* Sars, 1851**
–	First chaetiger uniramous; dorsally protruded neuropodial lobes absent in anterior abdomen; posteriorly extended notopodial lobes present in posterior abdomen	**2**
2	Lateral organs protruded above surface in anterior abdominal region	**3**
–	Lateral organs not protruded above surface in anterior abdominal region	**4**
3	Palpode indistinct; posterior abdominal region with unilobed notopodial lobes; genital pores present between chaetigers 5–10; all segments stain green in MGSP	***N. tenuis* Moor, 1909**
–	Distinct palpode present; posterior abdominal region with bi-lobed notopodial lobes; genital pores present between chaetigers 8–12; chaetigers 1–6 and dorsum of abdomen stain green, chaetigers 7–10 stain blue in MGSP	***N. hemipodus* Hartman, 1945**
4	Eyespots present on posterior prostomium; genital pores present between chaetigers 3 or 5–10	***N. torquatus* Hutchings & Rainer, 1979**
–	Eyespots absent on prostomium; genital pores present between chaetigers 7–11; dorsum of chaetiger 7 and chaetiger 8–10 stain blue in MGSP	***N. koreanus* n. sp.**

## Supplementary Material

XML Treatment for Notomastus

XML Treatment for Notomastus koreanus
